# Influence of Mitroflow bioprosthesis structural valve deterioration on cardiac morbidity

**DOI:** 10.1186/s13019-019-0875-1

**Published:** 2019-03-18

**Authors:** Farhad Waziri, Zarmiga Karunanithi, Brian Bridal Løgstrup, Vibeke Hjortdal, Per Hostrup Nielsen, Steen Hvitfeldt Poulsen

**Affiliations:** 1 Department of Cardiology, Aarhus University Hospital, Palle Juul-Jensens Blvd. 99, 8200 Aarhus N, Denmark; 2 Department of Cardiothoracic and Vascular Surgery, Aarhus University Hospital, Palle Juul-Jensens Blvd. 99, 8200 Aarhus N, Denmark; 3 Department of Clinical Medicine, Aarhus University, Palle Juul-Jensens Blvd. 99, 8200 Aarhus N, Denmark

**Keywords:** Aortic valve replacement, Structural valve deterioration, Bioprosthesis, Mitroflow, Cardiac morbidity

## Abstract

**Background:**

This study investigated the extent and nature of cardiac morbidity and cause of mortality in patients with Mitroflow structural valve deterioration (SVD).

**Methods:**

A retrospective study was performed examining the medical records of patients who had received Mitroflow bioprosthesis between February 2001 and April 2014 and died during this period. A total of 211 patients were identified and included in the analyses. To determine the cause of mortality, cases were divided into three predefined groups: cardiovascular death due to SVD (group 1), cardiovascular death with no SVD (group 2) and non-cardiovascular death without SVD (group 3).

**Results:**

Overall mortality in this study was 7.6% at 1 year, 46.4% at 5 years and 97.2% at 10 years. In group 1, 53 patients (25%) died; in group 2, 59 patients (28%) died; and in group 3, 99 patients (47%) died. Hospitalisation for congestive heart failure was observed in 49.1% in the SVD group vs. 10.2 and 13.1% in the two other groups, *p* < 0.001. Hospitalisation for endocarditis was also significantly higher in the SVD group (11.3%) than in the two other groups (6.8 and 0%), *p* < 0.05. Hospitalisation due to myocardial infarction, cerebral stroke, arrhythmia or other cardiac-related diseases was not significantly different between groups.

**Conclusion:**

Structural valve deterioration in Mitroflow bioprosthesis was associated with a high prevalence of hospital admissions due to congestive heart failure and endocarditis. Patients with Mitroflow bioprosthesis should be systematically and routinely followed with echocardiography, and reoperation should be considered if SVD has developed.

## Introduction

Aortic valve replacement has improved life expectancy in patients with severe aortic valve stenosis. Bioprosthetic as opposed to mechanical valves are mainly preferred in patients > 65 years, at which age shorter durability is thought to be counterbalanced by freedom from anticoagulation [1]. The Carpentier-Edwards (CE) Perimount (Edwards Lifesciences, Irvine, CA, USA) and the Mitroflow (LivaNova Group Inc., Vancouver, Canada) pericardial aortic bioprosthesis are commonly used worldwide [1–5]. Previous studies show satisfactory mid- and long-term results for both valves [1–5]. However, recent studies have reported early structural valve deterioration (SVD) in the Mitroflow valve compared with the Carpentier-Edwards (CE) Perimount valve [6]. Some studies show a pattern of SVD already 4 years after Mitroflow implantation [7, 8]. This is supported by a recent registry-based study which observed increased reoperation rates for Mitroflow prostheses size 19 and 21 compared with Carpentier-Edwards (CE) Perimount 19 and 21 mm valves [6].

Previous studies concerning Mitroflow valves have focused on all-cause mortality and showed increased crude mortality in Mitroflow bioprosthesis compared with other valve types. However, cause of death related to known SVD in Mitroflow bioprosthesis and the impact of living with a dysfunctional Mitroflow bioprosthesis on cardiac morbidity remain unclear. Therefore, further investigation is needed to clarify the influence exerted by the Mitroflow valve on cardiac morbidity and cause of mortality.

The aim of the present study was to investigate the extent and nature of cardiac morbidity and cause of mortality in patients with Mitroflow structural valve deterioration, and discuss relevant treatment options.

## Materials and methods

**Fig. 1:**
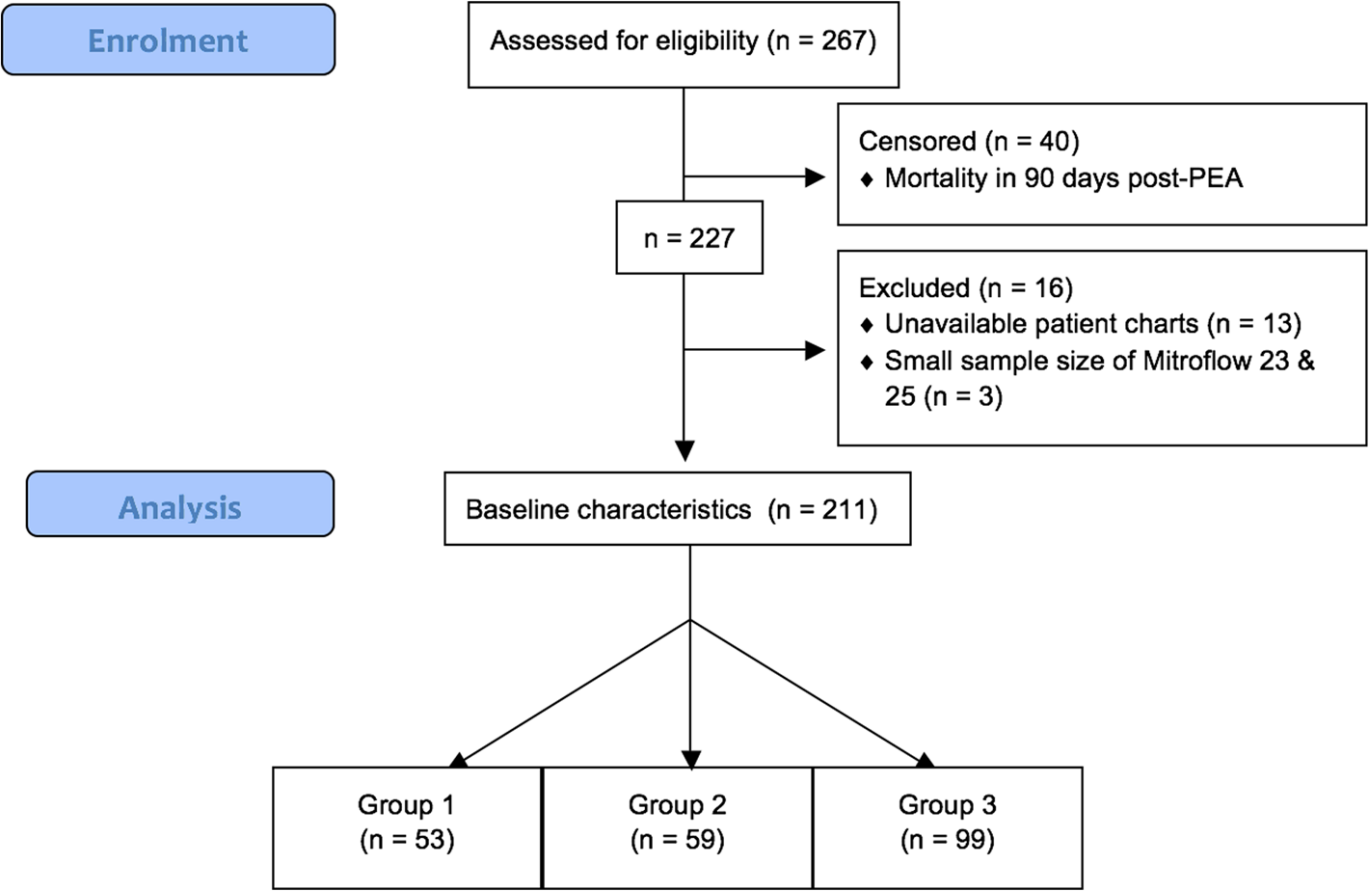
Enrolment of patients who had received Mitroflow bioprosthesis and died within the study period (February 2001 – April 2014). Group 1: Cardiovascular death due to SVD; Group 2: Cardiovascular death with no SVD; Group 3: Non-cardiovascular death without SVD

We conducted a retrospective population-based cohort study that included all patients who died after a Mitroflow pericardial bioprosthesis implantation within the study period. The patients were selected from a single centre, Aarhus University Hospital, Denmark, between February 2001 and April 2014. A total of 440 patients who received aortic valve replacement with the Mitroflow bioprosthesis were identified from the Western Denmark Heart Registry [9]. Since all Danes have a unique identification number, the registry provided a complete follow-up for all of the patients who received a Mitroflow bioprosthesis [10]. A total of 267 patients had died at the end of the inclusion period. We censored the first postoperative 90 days for confounding issues regarding operation complications to be able to clearly relate morbidity and mortality to subsequent SVD. On this basis, a total of 227 patients were left in the study (Fig. 1).

Morbidity was defined as either cardiac or non-cardiac diseases. Cardiac morbidities included congestive heart failure, endocarditis, stroke, myocardial infarction, supraventricular and ventricular arrhythmia; the remaining cardiovascular diseases were called “other cardiac diseases”. Non-cardiac morbidities, viz. infection, respiratory disease and cancer, were defined as “other non-cardiac diseases”. Morbidity data were collected from patient charts. Due to missing patient charts, 13 patients were excluded from the analysis. Due to the small sample sizes of Mitroflow 23 (*n* = 2) and size 25 (*n* = 1), these cases were also excluded from the study. Consequently, a total of 211 patients with either Mitroflow valve size 19 or 21 were included in the morbidity and cause of mortality analyses (Fig. 1).

To determine the cause of mortality, the cases were divided into three predefined groups: cardiovascular death due to SVD (group 1), cardiovascular death with no SVD (group 2) and non-cardiovascular death without SVD (group 3).

The study was approved by the Danish National Health Board (ref no: 1–16–02-527-16) and the Danish Data protection Agency (ref. no: 2012-58-006).

### Definition of SVD

SVD was defined by echocardiography as significant aortic valve prosthesis stenosis when the aortic valve area was below < 1.0 cm^2^, calculated from the continuity equation or from direct planimetry using transoesophageal echocardiography. Assessment of prosthesis stenosis was also based on the velocity ratio between the peak velocity in the left ventricle outflow tract and peak velocity across the prosthetic valve. A velocity ratio < 0.25 was considered as significant stenosis. In the presence of colour Doppler, SVD was considered as severe aortic insufficiency (vena contracta > 6 mm) along with volume overload on the left ventricle. Moderate aortic stenosis with an aortic valve area between 1 and 1.5 cm^2^ or a velocity ratio of 0.25–0.3 combined with moderate aortic regurgitation was also considered as significant SVD. The information was gathered from echocardiographic recordings.

## Statistical analysis

Descriptive data are expressed as mean ± standard deviation for continuous variables and proportions for categorical variables. Time-to-event was calculated from 90 days after the operation until the date of death. The overall incidences are illustrated in graphs. Due to competing risk, cumulative incidence functions were performed to evaluate the incidence of SVD. Cumulative incidence functions were calculated at year 5 and 10. Hospitalisation due to cardiac co-morbidity was analysed with Fisher’s exact test. Statistical analyses were performed using STATA version 14.2 (StataCorp. 2015). A *p*-value < 0.05 was considered significant.

## Results

### Patient characteristics

A total of 211 patients were included. Preoperative patient characteristics are outlined in Table 1. The mean age at operation was 77 ± 2 years (range: 62–94 years) and 155 (73.5%) were females. The mean follow-up time from 90 days after operation until death was 5 ± 2.8 years. A total of 1064.9 patient years of evaluation was calculated. The size of the Mitroflow was distributed as follows: size 19 (30.3%) and 21 (69.7%). The indication for Mitroflow bioprosthesis implantation in 208 (98.6%) patients was aortic stenosis; in the rest (*n* = 3, 1.4%), it was endocarditis. Pre-operatively, 64% of the patients received diuretics, 71% aspirin, 47% beta-blockers, 29% ACE/ATII-inhibitors, 43% statins, 24% calcium antagonists, 12% vitamin-K antagonists and 3% amiodarone. During Mitroflow implantation, 47% received concomitant coronary artery bypass grafting. The three defined subgroups used for morbidity and cause of mortality analyses had the same average age (77 years). A total of six patients (2.8%) received reoperation during the follow-up time, where three patients had endocarditis and the remaining three had aortic stenosis.

**Table 1:** Patient characteristics

	Total	Group 1	Group 2	Group 3	*P*-value
	*n* = 211	*n* = 53	*n* = 59	*n* = 99	
Age at operation (years)	76.8 ± 5.3	76.9 ± 5.7	76.9 ± 5.3	76.8 ± 5.2	0.992
Women	155 (73.5)	42 (79.3)	42 (71.2)	71 (71.7)	0.543
BSA (m^2^)	1.73 ± 0.19	1.72 ± 0.16	1.76 ± 0.21	1.72 ± 0.19	0.217
EuroScore II	1.6 ± 1.3	1.6 ± 1.1	1.6 ± 1.1	1.6 ± 1.6	0.999
NYHA	2.5 ± 0.7	2.5 ± 0.8	2.6 ± 0.8	2.4 ± 0.7	0.552
CCS	1.2 ± 1.3	1.2 ± 1.3	1.4 ± 1.2	1 ± 1.3	0.141
Blood pressure					
Systolic (mmHg)	147 ± 27	150 ± 28	144 ± 25	147 ± 27	0.574
Diastolic (mmHg)	79 ± 16	80 ± 20	77 ± 13	79 ± 15	0.730
eGFR (mL/min/1.73 m^2^)	50 ± 24	46 ± 18	52 ± 25	51 ± 25	0.574
≧50	53 (44.9)	11 (39.3)	19 (47.5)	23 (46)	0.783
< 50	65 (55.1)	17 (60.7)	21 (52.5)	27 (54)	0.783
Smoker					0.503
Never	68 (32.5)	21 (40.4)	17 (28.8)	30 (30.6)	
Active	95 (45.5)	19 (36.5)	31 (52.5)	45 (45.9)	
Quit	46 (22)	12 (23.1)	11 (18.6)	23 (23.5)	
Diabetes	26 (12.4)	7 (13.5)	6 (10.2)	13 (13.1)	0.830
Hypertension	115 (55)	33 (63.5)	34 (58.6)	48 (48.5)	0.173
Ischaemic heart disease	108 (51.9)	31 (59.6)	37 (63.8)*	40 (40.8)*	< 0.001
Previous PCI	9 (4.3)	4 (7.6)	3 (5.3)	2 (2.0)	0.261
Previous CABG	7 (3.4)	1 (1.9)	4 (6.8)	2 (2.1)	0.224
Extracardiac arteriopathy	23 (10.9)	7 (13.2)	10 (17.0)	6 (6.1)	0.086
Atrial fibrillation	41 (19.8)	15 (28.3)	12 (20.7)	14 (14.6)	0.13
Apoplexia cerebri	22 (10.6)	7 (13.2)	6 (10.3)	9 (9.4)	0.765
COPD	37 (17.9)	6 (11.5)	11 (18.6)	20 (20.8)	0.365
Pre-operative echo					
EF (%)	54 ± 13	53 ± 13	54 ± 12	55 ± 13	0.782
Aorta area (cm^2^)	0.68 ± 0.2	0.68 ± 0.2	0.68 ± 0.2	0.68 ± 0.2	0.968
Aorta CW max (mmHg)	74 ± 28	73 ± 28	70 ± 23	76 ± 32	0.486

Values are mean ± standard deviation or as number and percent

*BSA* Body surface area, *CABG* Coronary artery bypass graft, *CCS* Canadian Cardiovascular Society, *COPD* Chronic obstructive pulmonary disease, *EF* Ejection fraction, *eGFR* Estimated glomerular filtration rate, *PCI* Percutaneous coronary intervention, *NYHA* New York Heart Association

*Significantly different, *p* < 0.05

### Morbidity

Univariate analysis was performed to identify preoperatively clinical or echocardiographic variables that could predict later SVD development in patients with Mitroflow bioprosthesis. Univariate analysis showed no predicting variables for the development of SVD, p = NS (Table 2).

**Table 2:** Univariate analysis to identify preoperatively clinical and echocardiographic variables to predict development of SVD in patients with Mitroflow bioprosthesis

	Total (*n* = 211)		SVD (*n* = 53)	
	HR	*P*-value	HR	*P*-value
Mitroflow valve size 21	1.13	0.41	0.65	0.16
Sex (man)	1.19	0.27	1.13	0.72
Age (ref: 60–69 years)	1.00			
70–79 years	1.07	0.78	1.21	0.55
> 80 years	0.99	0.98	1.71	0.29
BSA (> 1.73 m^2^)	1.15	0.38	1.87	0.25
NYHA (ref: I)	1.00			
II	1.20	0.48	1.18	0.73
III	1.40	0.18	1.29	0.55
IV	2.10	0.06	NA	
EF (< 50%)	1.12	0.50	0.80	0.51
Smoker (ref: no)	1.00			
Previously	1.57	0.01	1.14	0.69
Active	1.41	0.08	1.30	0.48
Diabetes (yes)	0.99	0.95	1.18	0.69
IHD (yes)	1.07	0.61	0.99	0.97
Hypertension (yes)	0.96	0.79	1.10	0.73

*BSA* Body surface area, *NYHA* New York Heart Association, *EF* Ejection fraction, *IHD* Ischemic heart disease

**Fig. 2:**
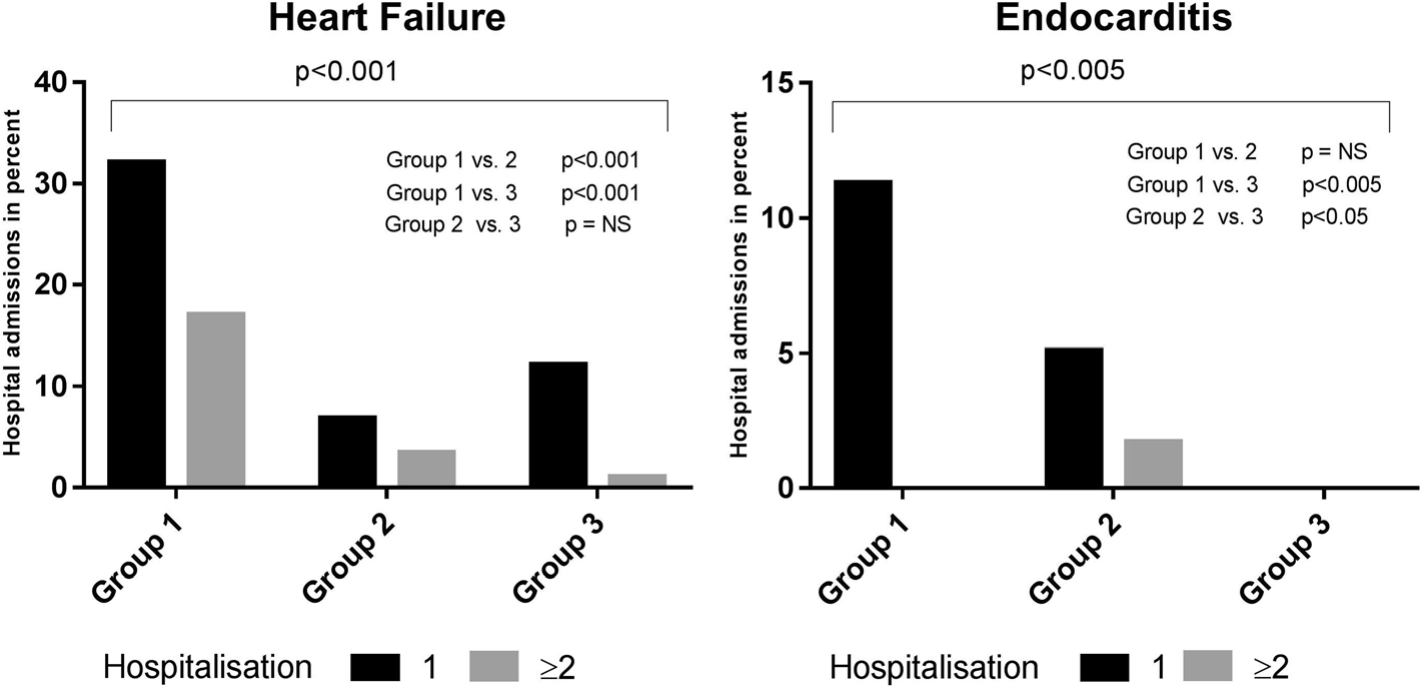
SVD-related complications. Percentage of hospital admissions with heart failure and endocarditis within the three different groups. Group 1: Cardiovascular death due to SVD; Group 2: Cardiovascular death with no SVD; Group 3: Non-cardiovascular death without SVD

**Fig. 3:**
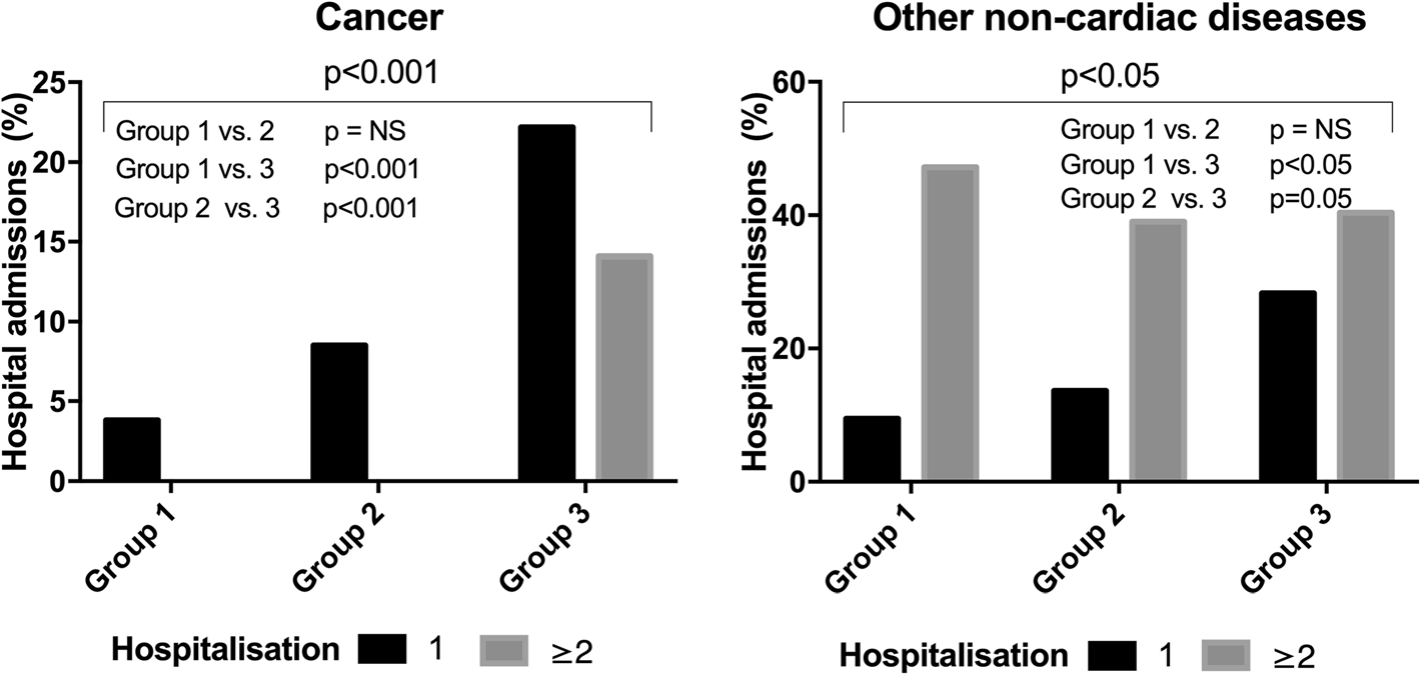
Kaplan Meier curve for all-cause mortality. Group 1: Cardiovascular death due to SVD; Group 2: Cardiovascular death with no SVD; Group 3: Non-cardiovascular death without SVD. P = NS

Of the total study population (*n* = 211), 53 patients (25.1%) developed SVD (group 1) during the follow-up period (13 years). The SVD group (group 1) had the largest hospitalisation rate for cardiac morbidity. In the pre-defined cardiac morbidity diseases, we observed the following: hospitalisation for congestive heart failure was observed in 49.1% of the patients in the SVD group (group 1) vs. 10.2% in group 2 and 13.1% in group 3, *p* < 0.001. Hospitalisation for endocarditis was also significantly higher in group 1 (11.3%) than in group 2 (6.8%) and group 3 (0%), *p* < 0.05 (Fig. 2). Hospitalisation due to myocardial infarction, cerebral stroke, arrhythmia or other cardiac-related diseases was not significantly different between the groups. Hospitalisation for cancer was lower in group 1 (3.8%) than in group 2 (8.5%) and group 3 (36.3%), *p*<0.001. Hospitalisation for other non-cardiac diseases was significantly higher in group 3 (68.7%) than in group 1 (56.6%) and group 2 (52.6%), *p*<0.05 (Fig. 3).

### Mortality

**Fig. 4:**
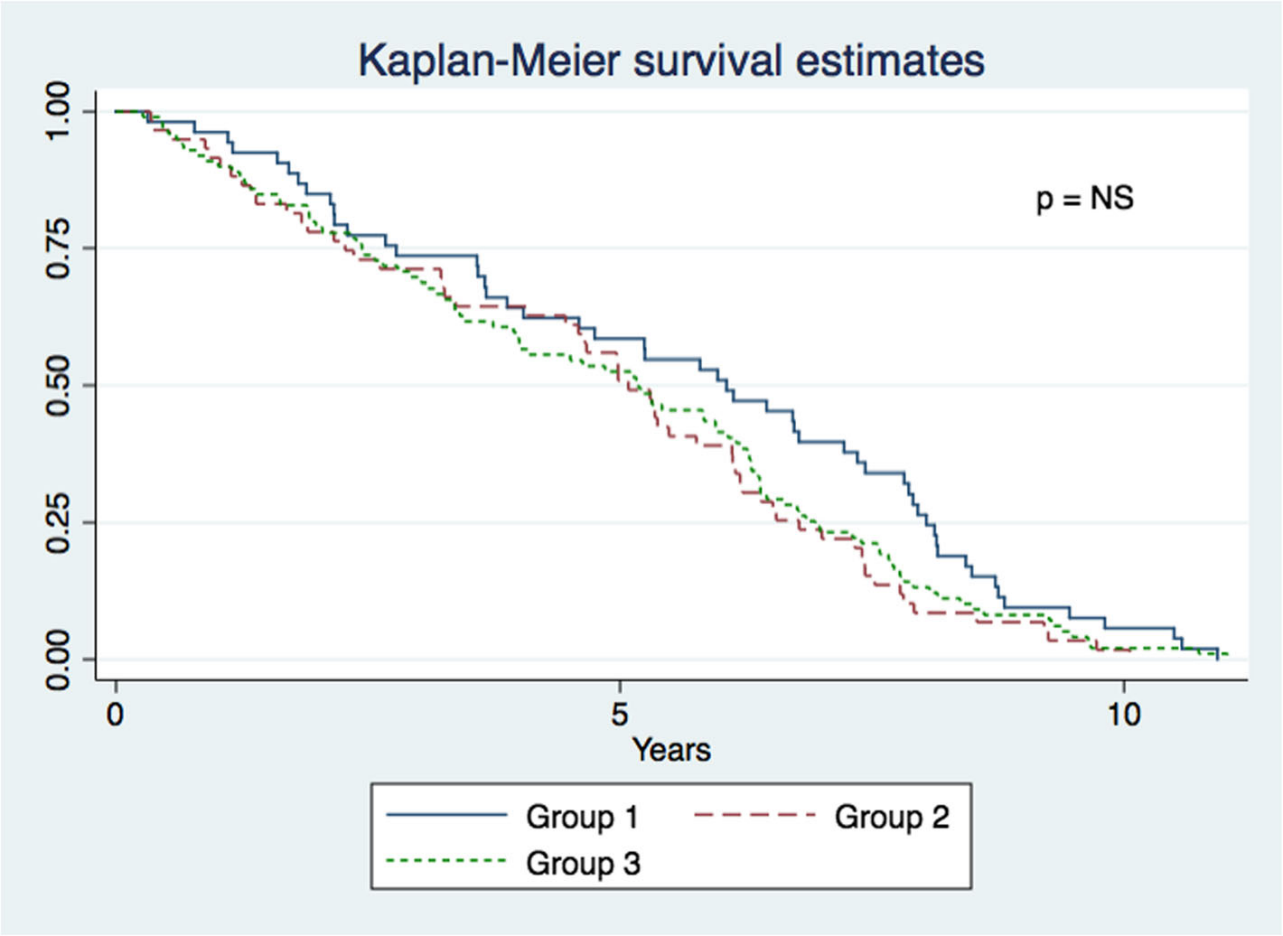
Cause-specific mortality. Mortality is defined by the three different groups. Group 1: Cardiovascular death due to SVD; Group 2: Cardiovascular death with no SVD; Group 3: Non-cardiovascular death without SVD

**Fig. 5:**
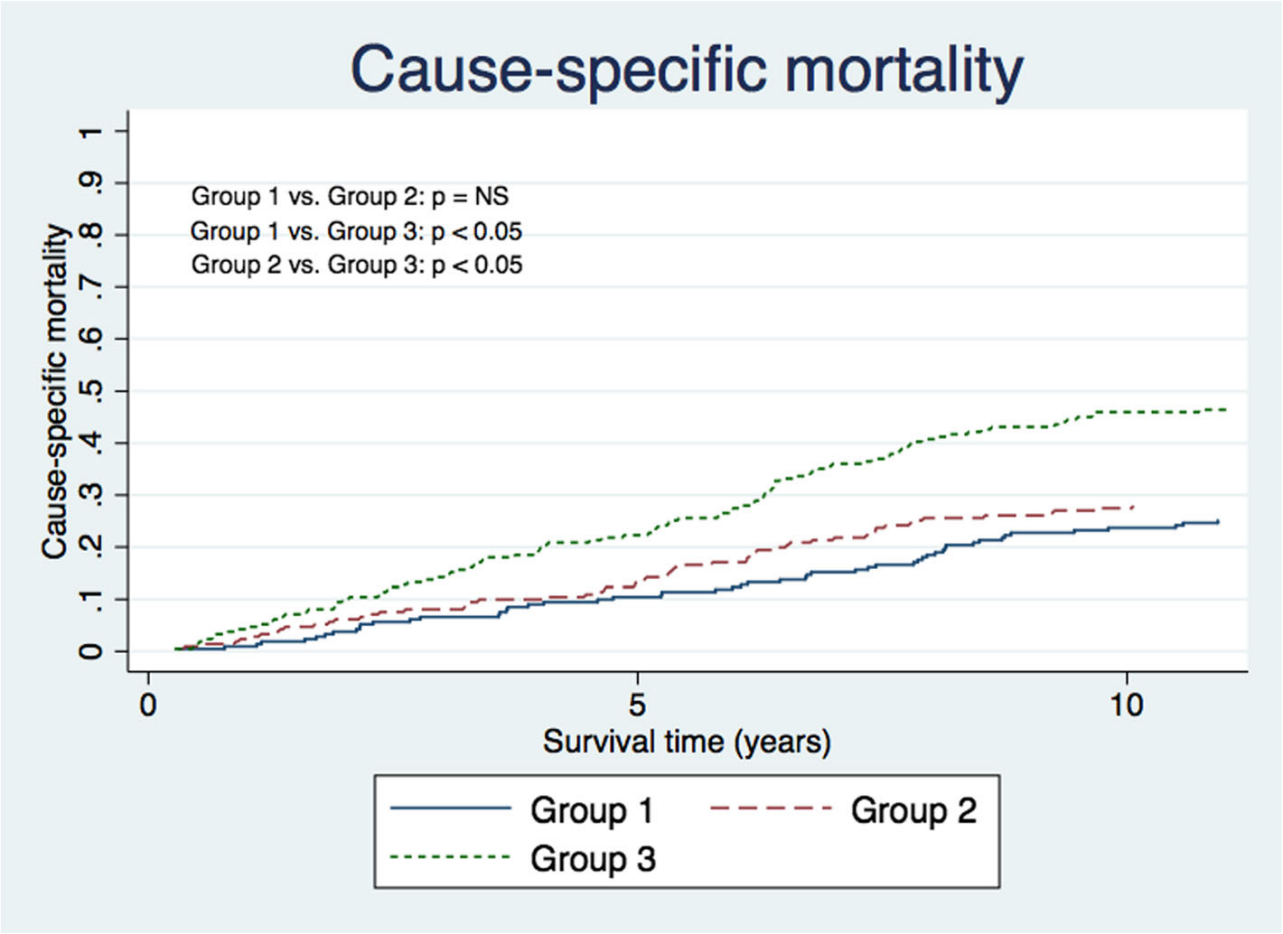
Non-cardiovascular death without SVD-related complications. Percentage of hospital admissions with cancer and non-cardiac diseases within the three different groups. Group 1: Cardiovascular death due to SVD; Group 2: Cardiovascular death with no SVD; Group 3: Non-cardiovascular death without SVD

During the follow-up time (13 years), 211 patients died after Mitroflow bioprosthesis implantation. In group 1, 53 patients (25%) died from SVD, in group 2, 59 patients (28%) died from cardiac disease without SVD, and in group 3, 99 patients (47%) died from non-cardiac disease without SVD. The overall mortality in this study was 7.6% at 1 year, 46.4% at 5 years and 97.2% at 10 years. No significant difference in all-cause mortality was found between groups 1, 2 and 3 (Fig. 4). The cumulative incidences at 5 and 10 years showed no significant difference in cause of mortality between groups 1 and 2. The cumulative incidence for groups 1 and 2 at 5 years was 9.5% (4.8; 14.3) vs. 12.2% (6.9; 17.5), and at 10 years 21.8% (15.1; 28.4) vs. 29.7% (18.5; 40.1), respectively. However, the cause-specific mortality in group 3 at 5 years was 13.6% (95% CI: 5.3; 21.9) and at 10 years 17.3% (95% CI: 3.6%; 31.0%) higher than in group 1, *p* < 0.05 (Fig. 5).

The cause-specific mortality analysis due to SVD was compared for Mitroflow 19 and 21 at 5 and 8 years. We observed a slightly higher but statistically insignificant risk of mortality among patients with a Mitroflow 19 compared with Mitroflow 21 (*p* < 0.4).

## Discussion

In the present study, we describe for the first time cardiac morbidity and cause of death in a patient population that had received a Mitroflow bioprosthesis implantation. In particular, we identified patients who developed SVD in the Mitroflow bioprosthesis and compared their cardiac-related morbidity and cause of death with that of patients without signs of SVD.

This study demonstrated that patients with SVD had a significantly higher hospital admission rate due to heart failure and endocarditis than patients who died from cardiovascular events without SVD and patients who had a non-cardiac death without SVD. It is well known that heart failure and endocarditis are serious complex conditions associated with reduced survival, requiring long and often repeated hospital stays and impaired quality of life. A total of 53% of patients with SVD were admitted with congestive heart failure, compared with 10% for group 2 and 13% for group 3. Acute admission with congestive heart failure often requires intensive care with intravenous diuretic and inotropic treatment. In-hospital stays are often extended by close follow-ups in an outpatient clinic, with substantial social and economic costs. Furthermore, our study shows that patients with congestive heart failure are often re-admitted (35%).

We know from other studies that congestive heart failure is associated with poor prognosis [11–13]. Randomised controlled studies with beta-blockers and angiotensin-converting enzyme treatment show increased mortality in congestive heart failure patients if they are not treated [14–17]. Therefore, systematic follow-up after Mitroflow implantation with transthoracic echocardiography is necessary to identify SVD at early stages and prevent the development of congestive heart failure. However, this study showed no increased cardiac mortality in the SVD group compared with the non-SVD groups. One explanation could be early detection and treatment of SVD-related complications; another possible explanation could be the small population size, which is a limitation of our study.

Hospitalisation due to endocarditis after Mitroflow bioprosthesis implantation was mostly associated with the SVD group. Prosthetic heart valve endocarditis often requires lengthy intensive care with cardiac monitoring, 4–6 weeks of intravenous antibiotics and, in some cases, reoperation of the aortic valve. Furthermore, heart valve prosthesis endocarditis is frequently associated with severe extracardiac infective manifestations. After protracted hospitalisation, patients require extensive cardiac rehabilitation. From a health and socioeconomic perspective, it can be assumed that both congestive heart failure and endocarditis are associated with substantial burdens for patients and society, which should be taken into account when evaluating SVD.

Contrary to our expectations, this study found no significant difference in all-cause mortality between any of the three groups. When we adjusted for cause of death, we found that, at 5 and 10 years, significantly more died in group 3 than in group 1 and group 2. The higher mortality in group 3 might be related to concurrent non-cardiac morbidities, such as cancer, that naturally increased the death rate. Even though patients in group 1 were much more frequently re-admitted postoperatively due to heart failure and endocarditis, these conditions did not seem to translate into a higher mortality rate compared with patients who died from cardiovascular conditions without SVD. However, the preoperative risk profile was similar. The differences in co-morbidities between the three groups complicated comparison between the groups. It would have been more accurate to compare the cause of death between the SVD group and the background population. Another limitation was the small size of the SVD group which also complicated comparison with groups 2 and 3.

Previous studies have demonstrated that age (below 70 years and above 80 years), Mitroflow valve size 19, ejection fraction below 35% and poor New York Heart Association functional class were independent risk factors for developing SVD [5, 18, 19]. Other studies have not identified small valve size as a risk factor [20]. However, the present study found no clinical variables that could predict the development of SVD. A possible explanation could be that the problem of developing SVD does not reside within the patient but in the structure of the Mitroflow valve itself [21]. In contrast to a previous case series [18], Lus F et al. found that the DLA Mitroflow model with its anticalcification treatment did not emerge as a protective factor against SVD in their study [20]. However, it has been mentioned that the pathologic mechanisms of SVD, concerning the degenerated LA/LXA Mitroflow prostheses shows more often calcification than cusp tear, in accordance with the results reported by Luk et al. [21]. Patients with a degenerated Mitroflow prosthesis developed prosthesis stenosis more often than regurgitation. This fact has important clinical consequences since patients with SVD withstand more easily a pressure than a volume overload. This is also in agreement with our long-term observations regarding more hospitalizations for congestive heart failure caused by the pressure overload observed.

We suggest that patients with Mitroflow bioprosthesis are systematically followed postoperatively with transthoracic echocardiography since SVD is related to severe morbidity and has financial consequences for patients and hospitals. Early detection of SVD could prevent related morbidities and reduce socioeconomic costs. Since most of the patients are elderly, reoperation of the aortic bioprosthesis is often not recommended due to the high risk of mortality. An alternative treatment is a valve-in-valve implantation. Valve-in-valve treatment has been reserved mainly for bioprosthesis size 21 and above, but several studies have had success with fracturing the ring of a small Mitroflow bioprosthesis by high-pressure balloon predilatation and then allowing for valve-in-valve treatment [22–25].

## Limitations

We acknowledge the limitation that the present study reflects the experiences of a single centre with a limited number of patients. Another limitation is that this study only assesses the smaller Mitroflow valve sizes 19 and 21, so we cannot conclude anything about larger size valves. However, this study has a great strength as the registries allowed for complete follow-up. Additionally, patients were included consecutively from a single centre, which might minimise differences in pre-, peri- and postoperative care.

## Conclusion

Structural valve deterioration in Mitroflow bioprosthesis was associated with a high prevalence of hospital admissions due to congestive heart failure and endocarditis. SVD developed in 25% of the total study population. Patients with SVD died from cardiac-related complications. The findings of this study have a number of important implications for future practice. Firstly, patients with Mitroflow bioprosthesis should be systematically and routinely followed with echocardiography; and secondly, reoperation should be considered if SVD has developed. Especially, the possibility of a valve-in-valve strategy with transcatheter aortic valve implantation should be considered.

## Abbreviation

SVD: Structural valve deterioration
